# Coverage and Characteristics of the Affymetrix GeneChip Human Mapping 100K SNP Set 

**DOI:** 10.1371/journal.pgen.0020067

**Published:** 2006-05-05

**Authors:** Dan L Nicolae, Xiaoquan Wen, Benjamin F Voight, Nancy J Cox

**Affiliations:** 1 Department of Statistics, The University of Chicago, Chicago, Illinois, United States of America; 2 Department of Human Genetics, The University of Chicago, Chicago, Illinois, United States of America; 3 Department of Medicine, The University of Chicago, Chicago, Illinois, United States of America; University of Michigan, United States of America

## Abstract

Improvements in technology have made it possible to conduct genome-wide association mapping at costs within reach of academic investigators, and experiments are currently being conducted with a variety of high-throughput platforms. To provide an appropriate context for interpreting results of such studies, we summarize here results of an investigation of one of the first of these technologies to be publicly available, the Affymetrix GeneChip Human Mapping 100K set of single nucleotide polymorphisms (SNPs). In a systematic analysis of the pattern and distribution of SNPs in the Mapping 100K set, we find that SNPs in this set are undersampled from coding regions (both nonsynonymous and synonymous) and oversampled from regions outside genes, relative to SNPs in the overall HapMap database. In addition, we utilize a novel multilocus linkage disequilibrium (LD) coefficient based on information content (analogous to the information content scores commonly used for linkage mapping) that is equivalent to the familiar measure *r* ^2^ in the special case of two loci. Using this approach, we are able to summarize for any subset of markers, such as the Affymetrix Mapping 100K set, the information available for association mapping in that subset, relative to the information available in the full set of markers included in the HapMap, and highlight circumstances in which this multilocus measure of LD provides substantial additional insight about the haplotype structure in a region over pairwise measures of LD.

## Synopsis

The ability to survey hundreds of thousands of single nucleotide polymorphisms (SNPs) with cost-effective technologies is enabling investigators to conduct genome-wide association studies designed to find genetic variation affecting disease risk. To facilitate both interpretation of these studies and the design of follow-up studies, Nicolae and colleagues have made a comprehensive survey of the distribution and coverage of the first of these high-throughput platforms for genome-wide association mapping to be made publicly available, the Affymetrix GeneChip Human Mapping 100K set of SNPs (100K set). They found that SNPs within coding sequence are underrepresented in this mapping set relative to the set of SNPs included in the International HapMap Project, and this has consequences for the success of association studies. Measuring the information content confirms that the 100K set provides substantial coverage on variation in the HapMap database. The 100K set is quite redundant, as the SNPs were selected in the absence of information on the correlation (linkage disequilibrium) among them, and thus the relatively high value of the information content in the 100K set for the HapMap SNPs bodes well for general ability to survey genomic variation with a subset of variants.

## Introduction

There has been widespread anticipation of the identification and characterization of susceptibility loci for complex phenotypes through genome-wide association mapping since Risch and Merikangas [1] noted the potential for both the practical development of methods of genotyping for such studies and the increased power for detecting susceptibility loci with modest to moderate effects promised by these high-resolution maps. There are a wide variety of studies already completed and currently under way using currently available technologies, including the Affymetrix GeneChip Human Mapping 100K single nucleotide polymorphism (SNP) set [2] (hereinafter referred to as the 100K set), as well as approaches that are now undergoing large-scale testing. Because there have already been successes using genome-wide association mapping [3,4], more extensive use of these approaches seems likely.

The development of affordable high-throughput technologies for genome-wide association mapping reflects the concomitant increase in the publicly available information on polymorphisms in the human genome [5–7] and is clearly made more valuable by the emerging data on haplotypes and their frequencies as well as the extent of linkage disequilibrium (LD) in human populations that is available through the HapMap initiative [8]. Thus, it is both possible and desirable to utilize these genomic resources now to inform our understanding of the coverage and characteristics of the SNP sets that are proposed for genome-wide association mapping.

Much of the research to date in characterizing the LD among SNPs has utilized pairwise measures of LD to characterize the extent to which any set of SNPs captures the information present in a larger set of polymorphisms. The recent development of a multilocus measure of LD [9], analogous to the pairwise measure *r* ^2^ that is most commonly considered in the context of study design for association mapping, provides a more comprehensive assessment of LD in a region, particularly in the context of such relative comparisons. Here we provide an analysis of the extent to which SNPs available for genome-wide association mapping summarize the potential information available in the entire set of HapMap SNPs for the CEU (Utah residents with ancestry from northern and western Europe) and YRI (Yoruba people in Ibidan, Nigeria) HapMap data, with respect both to the coverage and the characteristics of the polymorphisms.

Although there are, of course, many ways to characterize a set of SNPs, we focus here on classifications reflecting the potential function and location of polymorphisms relative to genes and coding sequences: nonsynonymous, or synonymous; within an intron, mRNA UTR, or within 2 kb of a gene (locus region), or it could be a change in an intron/exon splice junction. SNPs belonging to more than one category are indicated as being in “multiple classes” and are generally within genes, but fall into more than one functional classification. If no functional information is present in dbSNP (i.e., if the SNP is more than 2 kb from a known dbSNP gene), we defined the SNP to be “nongenic.”

The goal of this investigation was to characterize the pattern and distribution of SNPs in the Affymetrix 100K set with reference to the information in the HapMap set, which was the only SNP set proposed for genome-wide association, and for which the list of included SNPs was publicly available and fully included in the Phase I release of the HapMap database.

## Results

Table 1 shows the frequencies of SNPs in the HapMap [8] Phase I public release #16a, the Perlegen database [10], and in the 100K set in the categories described above, defined by the position of the SNPs relative to genic and nongenic regions. There is an increase in the proportion of intergenic 100K-set SNPs relative to the HapMap and Perlegen proportions, compensated by a decreased proportion of markers located in gene exons. To determine if this distribution of SNPs is related to the chromosomal density of the arrayed variation, we plotted the 100K set–SNP density versus the HapMap density of markers for each chromosome (Figure 1A). Chromosomes 19 and 22 have a much lower density of 100K SNP set markers than chromosomes with a similar HapMap density. We note that Chromosomes 19 and 22 have higher proportions of SNPs in genes (coding and noncoding parts) than other chromosomes. Thus, the finding of reduced 100K SNP density relative to HapMap SNP density on Chromosomes 19 and 22 is consistent with our overall observation that SNPs in the 100K set are undersampled from coding regions and oversampled from regions outside genes. We found that there is a significant positive correlation (*ρ* = 0.82) between the 100K SNP–set density and the proportion of HapMap SNPs more than 2 kb from a gene. There is a similar negative (*ρ* = −0.8) correlation (Figure 1B) between the density of SNPs in the 100K SNP set and the proportion of SNPs in coding regions (synonymous and nonsynonymous). The deficiency of both synonymous and nonsynonymous categories of SNPs in the 100K SNP set relative to the HapMap SNP set appear in most autosomal chromosomes.

**Table 1:**
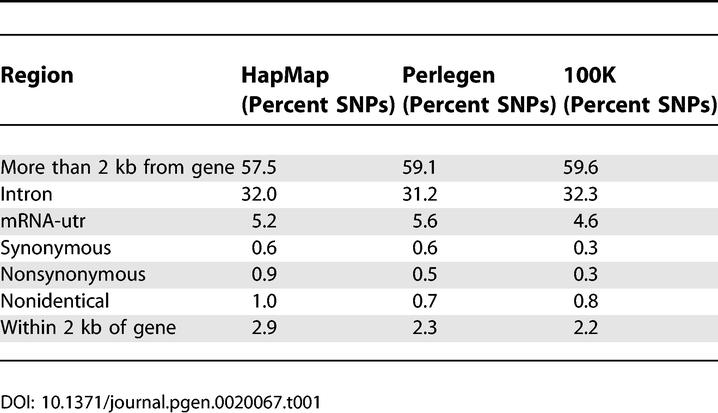
Regional Locations for Affymetrix Mapping 100K SNP Set, HapMap SNP Set, and Perlegen SNP Set

**Figure 1:**
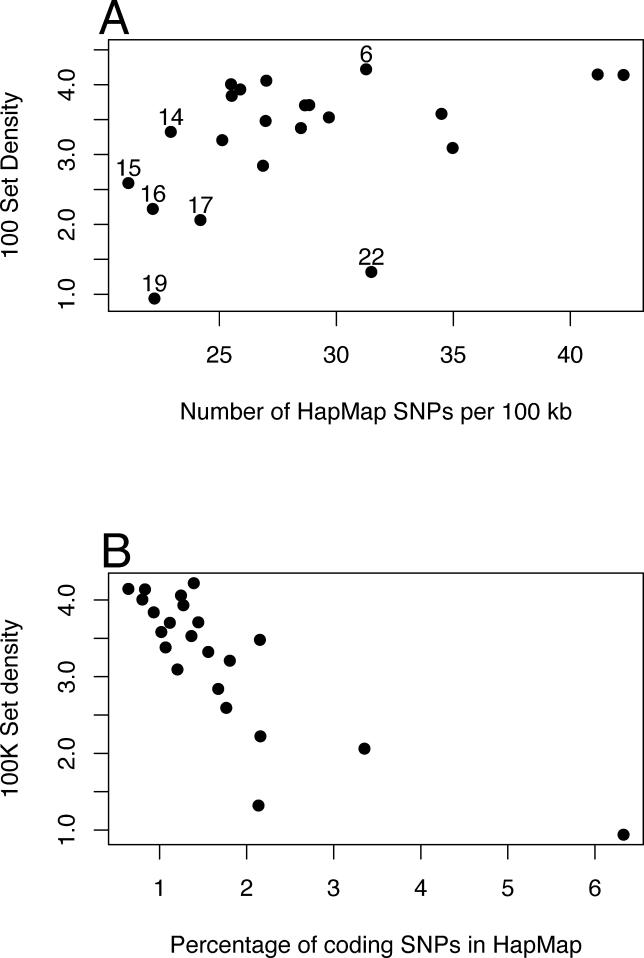
Factors Explaining the 100K Set Chromosomal Density Chromosomes mentioned in the text are labeled, as are chromosomes that have similar HapMap densities to those that are discussed. (A) Plots the density (number of SNPs/100 kbp) of the Affymetrix 100K set for the 22 autosomal chromosomes against the average number of SNPs/100 kb in the HapMap database. (B) Plots the density of the 100K set in each chromosome as a function of the percentage of HapMap SNPs in that chromosome located in exons (synonymous or nonsynonymous).

The selection of the SNPs for the 100K SNP set [2] was taken from a database of more than 3 million SNPs. After reducing the database to a half million SNPs using biochemical and bioinformatics constraints, the second step in the selection of SNPs was intended to yield an estimated minor allele frequency (MAF) larger than 2% (more than two minor frequency alleles present in the 108 chromosomes genotyped). As shown in Figure 2, the distribution of the MAFs (estimated from the HapMap data) of the SNPs in the 100K SNP set nevertheless has more mass on the low frequencies than SNPs in the overall HapMap set. Figure 3A shows the distribution of the measure of multilocus linkage disequilibrium, *M_D_,* for all the markers in the HapMap database, including the markers in the 100K SNP set (for which *M_D_* = 1). Note that the multilocus LD measure has the same interpretation as *r* ^2^; in particular, it is equal to 1 if there is perfect LD between the “target” SNP and a group of markers in the 100K set, and is equal to 0 if the “target” variant is in linkage equilibrium with the SNPs in the 100K set. The median value of the distribution is 0.632. Note that of the more than 250K SNPs in the HapMap SNP set that have high *M_D_* values, almost half are SNPs from the 100K SNP set. Figure 3B similarly displays the distribution of the maximum *r* ^2^ value between each marker in HapMap and the 100K SNP set markers within 200 kb of them. The histograms of max *r* ^2^ and *M_D_* have a similar shape, but the *M_D_* distribution has more mass in the rightmost bar and less mass in the leftmost bar, evidence that max *r* ^2^ doesn't capture fully the amount of information contained in the genotyped variation.

**Figure 2:**
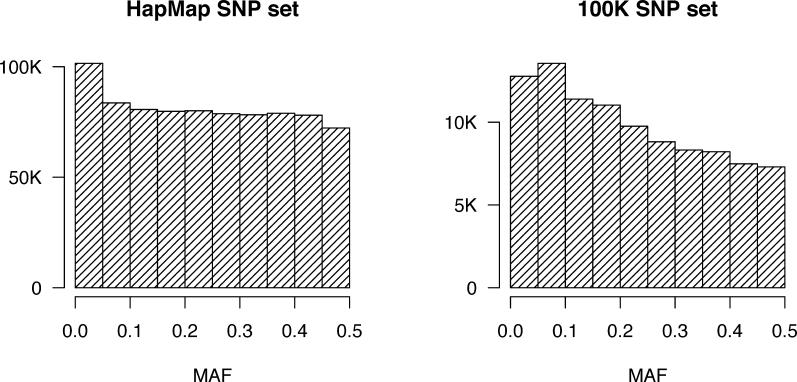
Histograms of MAF for HapMap and 100K SNP Sets

**Figure 3:**
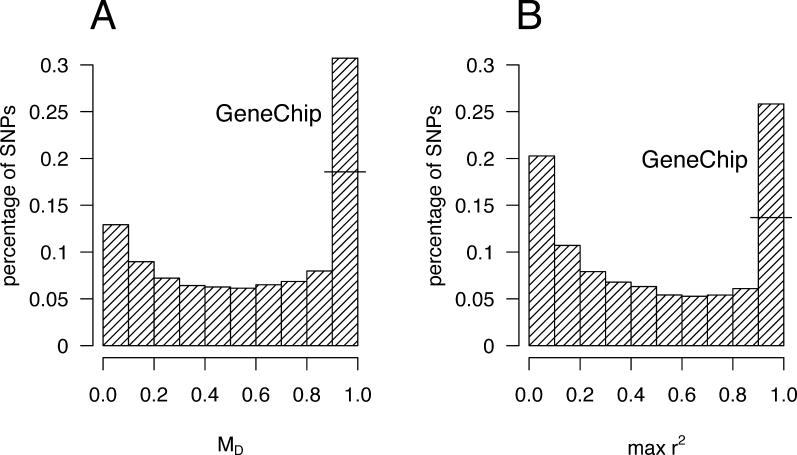
Information on HapMap Variation (A) Shows the distribution of the measure *M_D_* for all markers in the HapMap SNP set, including the markers in the Affymetrix 100K set. The part of the rightmost bar above the horizontal line corresponds to the markers in the 100K SNP set. (B) Displays a similar histogram for the maximum *r* ^2^ value between each marker in HapMap and the 100K SNP set markers within 200 kb of them.

As shown in Figure 4, more information is extracted from the 100K SNP set for HapMap SNPs in intergenic regions (where density on the 100K set is relatively higher) than for synonymous HapMap SNPs (where density on the 100K set is relatively lower).

**Figure 4:**
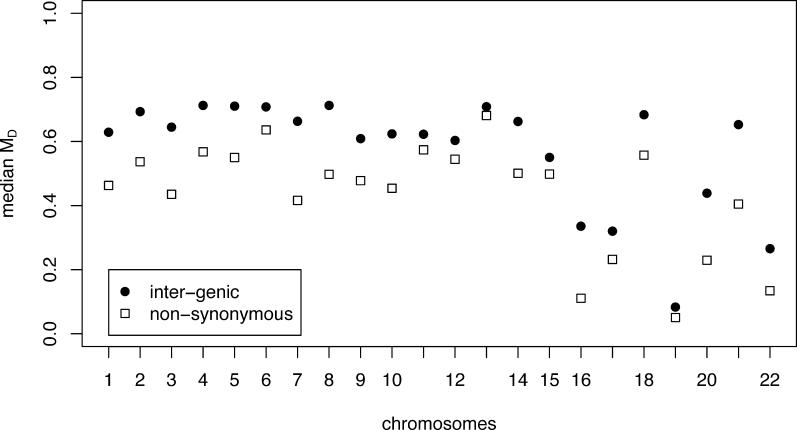
The Variability in Chromosomal Information Median value of the multilocus measure of LD. *M_D_* is plotted for each chromosome for intergenic SNPs located more than 2 kb from a gene (filled circles) and for nonsynonymous SNPs within exons (open squares).

Figure 5 illustrates the LD information summarized by *M_D_* and by the maximum of the pairwise values of *r* ^2^ for a 340-kb region on Chromosome 1 starting at rs666371 (20511030 bp on HapMap physical map). The filled triangles at the top of Figure 5 indicate the locations of the nine 100K SNPs in this region. For each HapMap marker, T, in the region that is not in the 100K set, both the multilocus measure of LD, *M_D_*(*T,*A*)* (filled circles), and a pairwise measure of LD calculated as the maximum of all pairwise values of *r* ^2^ between the test SNP and the 100K SNPs within a 200-kb region around T, denoted as max*_S_ r* ^2^(*T,S*) (open circles), were calculated. The region was chosen because it contains the first marker on Chromosome 1 (namely rs12753144) for which *M_D_*(*T,*A) − max*_S_ r* ^2^(*T,S*) > 0.9. Note that for many of the markers, the two ways of measuring LD (multilocus and pairwise) yield similar values, but there are several markers where the results differ greatly. Table 2 shows the haplotype frequencies for the set of markers containing rs12753144 (T in Table 2) and the four most informative markers in the 100K SNP set for interrogating allele frequencies at this marker. Note that the maximum of the pairwise values of *r* ^2^, max*_M_ r* ^2^ = 0.084, suggesting that virtually no information is available from the local markers for interrogating allele frequencies in cases and controls at this marker. In contrast, D′ between rs12753144 and any of the other markers is equal to 1 because there are only three distinct haplotypes present for each pairing of rs12753144 with another marker. Since tagging the *H*_1_ haplotype is sufficient for defining T (the rarer allele is found exclusively on this haplotype), the only loss of information comes from unknown phase. Note that this haplotype does not consist of consecutive markers.

**Figure 5:**
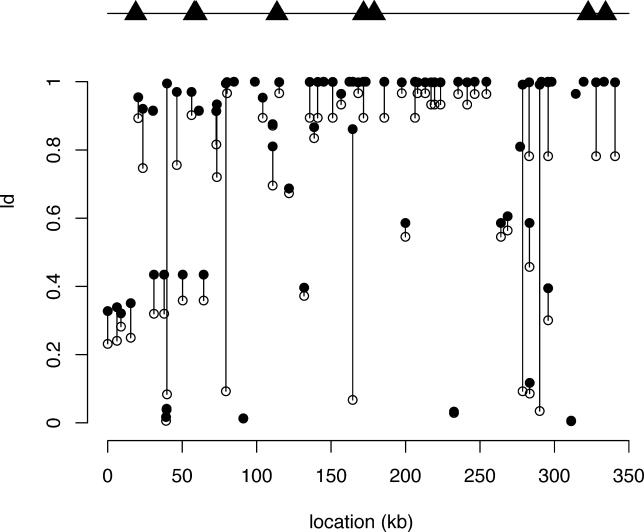
Differences in Pairwise and Multilocus LD Summary of LD estimated with the multilocus measure, *M_D_,* and maximum of the pairwise measure, *r* ^2^, for HapMap SNPs in a 370-kb region on Chromosome 1 starting at rs666371 (20511030 bp on HapMap physical map). The filled triangles at the top indicate the location of nine SNPs that are part of the 100K set. For each HapMap SNP, the value of *M_D_* was calculated using all 100K-set SNPs within 200 kb (filled circles), and similarly we calculated the maximum value of *r* ^2^ between the HapMap SNP and the same set of the array SNPs (open circles). The vertical lines connect the values of *M_D_* and max *r* ^2^ for each marker.

**Table 2:**
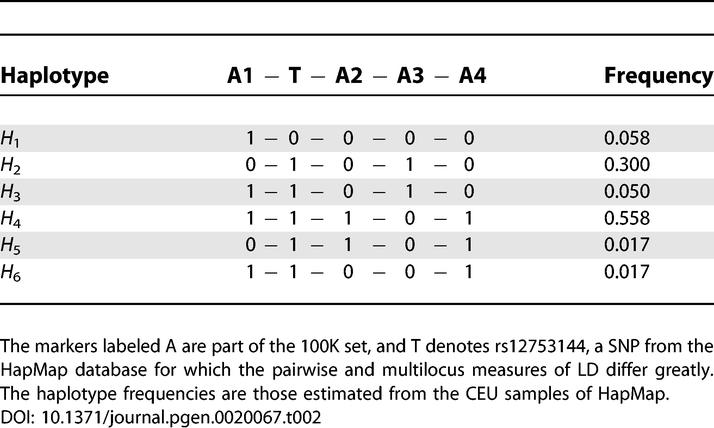
Estimated Haplotype Frequencies for Markers in the Region Shown in Figure 7

Similar to the calculations described above, we calculated, for each marker T on the 100K set, the information content *M_D_*(*T,*A), where the set A consists of the 100K SNP set in the region of T (but not including T), as shown in Figure 6. We comment on the interpretation of Figure 6 in the Discussion section.

**Figure 6:**
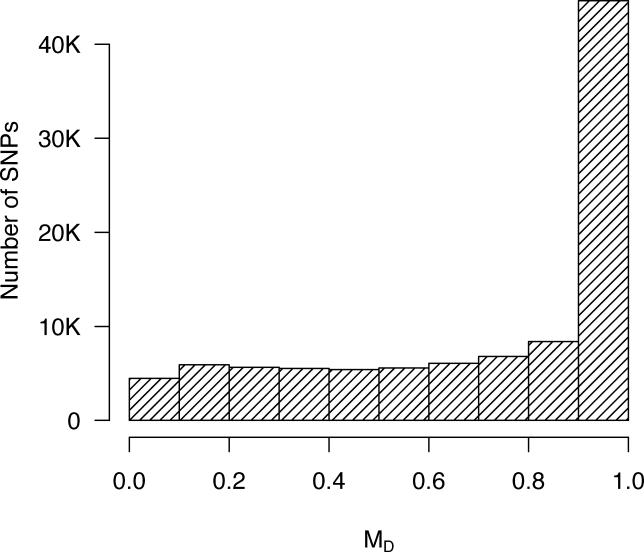
Histogram Summary of Information Content as Assessed by *M_D_* in the 100K SNP Set The value of *M_D_* was calculated for each SNP in the 100K SNP set using only the 100K SNPs from that region, but excluding the actual SNP under consideration.

**Figure 7:**
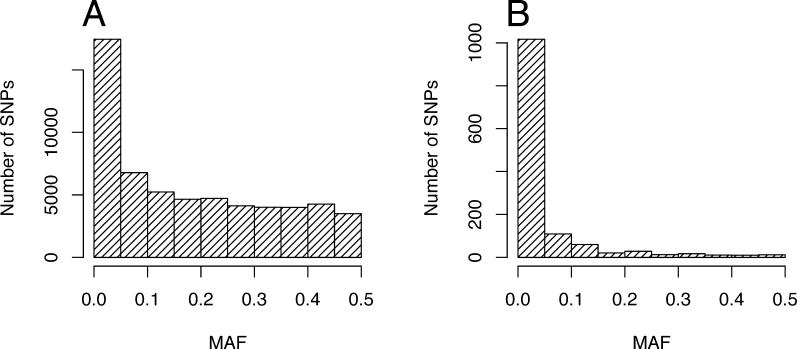
The Distribution of MAFs for Low-Information SNPs (A) Shows the histogram plots of the MAFs for all HapMap markers for which the 100K SNP set does not provide information (*M_D_* < 0. 05). (B) Shows subset of the markers described in (A) that have at least 10 SNPs from the 100K SNP set within 100 kb.

Figure 7A shows the distribution of MAF values for HapMap SNPs that are poorly interrogated (*M_D_* < 0.05) by the 100K SNP set. Figure 7B shows the distribution of MAF values for HapMap SNPs that are poorly interrogated (*M_D_* < 0.05) despite having at least 10 SNPs from the 100K SNP set within 100 kb. Figure 8 summarizes the difference in the information extracted by the 100K set in the CEU and YRI samples.

**Figure 8:**
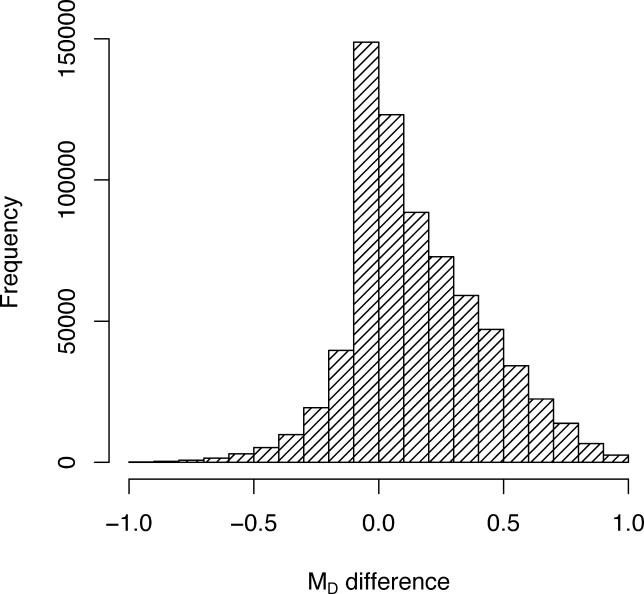
The Contrast between CEU and YRI LD Patterns Histogram of the difference between *M_D_* values calculated in the European HapMap and African HapMap samples (*M_D-European_ − M_D-African_*) when the Affymetrix 100K–SNP subset phased in both samples was used to provide information on the entire HapMap SNP set.

## Discussion

Overall, our analyses show that SNPs in the 100K set are undersampled from coding regions and oversampled from regions outside genes relative to the distribution of SNPs in the HapMap. Moreover, the lower proportion of SNPs in coding regions in the 100K SNP set relative to the HapMap and Perlegen SNP sets does not appear to reflect an oversampling of polymorphisms with higher MAFs; the distribution of the MAFs of the SNPs in the 100K SNP set actually has more mass on the low frequencies than SNPs in the overall HapMap set. The high correlation between the density of coding or noncoding SNPs in HapMap and the density of SNPs in the Affymetrix 100K SNP set suggest that the variants in the 100K set were not randomly chosen—the sequence constraints in choosing the probes have a severe consequence on the location of the chosen SNPs.

Nevertheless, the relatively high median value of the multilocus measure of LD, *M_D_,* of 0.632, calculated for all markers in the HapMap set (including the 100K set markers) confirm that typing a low proportion of the total variation can capture a large fraction of the information available. As might be expected, the lower the density of SNPs in a region, the less information is extracted. Thus, the information provided by the 100K SNP set on other HapMap polymorphisms is lower in functional regions of the genome, notably in exons, than in intergenic regions. Follow-up studies in genome-wide association mapping efforts using the 100K SNP set may utilize this type of information-content assessment to ensure more uniform interrogation of the variation in regions near genes with some signal in preliminary analyses.

Ideally, high-throughput platforms for genome-wide association mapping would have little redundant information. Unfortunately, there is considerable redundancy in the 100K SNP set; note that *M_D_* for more than 40,000 SNPs is near 1. This redundancy will no doubt decline in later-generation products that are able to incorporate information on LD derived from the HapMap. In the interim, knowledge of the redundancy can be put to good use in, for example, exploring genotyping discrepancies based on local-sequence context that might inform the design of later-generation products.

The amount of information extracted does of course depend on the MAF (Figure 7), and power calculations should take this into account. Among the HapMap SNPs that are poorly interrogated (*M_D_* < 0.05) by the 100K SNP set, SNPs with low MAF are prominently represented. But results of our studies in regions with many local 100K-set SNPs bode well for the ability of later-generation denser SNP sets to interrogate all common variation. We found that the distribution of MAF values for HapMap SNPs that are poorly interrogated (*M_D_* < 0.05), despite having at least 10 SNPs from the 100K SNP set within 100 kb, came exclusively from the rare frequency spectrum. Moreover, the multilocus linkage disequilibrium measure we use can give a very different assessment of the ability of a subset of SNPs to interrogate the allele frequencies in a larger set than the corresponding measures based on two-locus LD patterns. This is particularly useful information in determining how well the allele frequencies for each non-typed HapMap SNP can be interrogated using a defined set such as the 100K SNP set.

It is obvious that power calculations that assume the risk variant has an *r* ^2^ of, let's say, 0.6 to a genotyped variant ignore important characteristics of the genotyped set: the average amount of LD to an untyped SNP depends on the allele frequency and location (e.g., genic versus nongenic) of the SNP. For example, we have less power with the 100K set to find rare nonsynonymous than common intergenic risk factors. Relevant power calculation should take these characteristics into account and assign prior disease susceptibility probabilities on different groupings decided based upon allele frequencies and SNP annotation.

As would be expected from results of studies of LD in human populations [11–13], the 100K SNP set generally provides more information on the entire set of HapMap SNPs in the European than in the African samples. It should be noted, however, that although the European sample has higher values of *M_D_* than the African sample for most SNPs from the 100K set that are phased in both samples, an appreciable number of markers have a higher value of *M_D_* in the African than in the European sample.

Many of the obvious flaws in first-generation platforms for genome-wide association mapping will be improved in subsequent versions of the technology. Notably, taking LD among SNPs into account in SNP selection will substantially reduce redundancy and improve the interrogation of untyped variation, given a fixed number of SNPs. But the requirements for high-throughput platforms may preclude completely representative surveys of the genome for some time to come. The need to achieve high call rates leads to reduced representation of SNPs in regions with sequence similarity, including, at least for now, conserved regions within genes. Thus, it will be useful to conduct studies such as these to provide context for the interpretation of results of genome-wide association studies and information for the design of follow-up studies.

## Materials and Methods

We obtained from dbSNP (http://www.ncbi.nlm.nih.gov/SNP/index.html) build #23 information regarding the genic and nongenic nature of all referenced SNPs genotyped in the Phase I release of the HapMap data. For all SNPs, we extracted the physical positions (for genome build #4), strand orientation, and alleles for the given SNP. For SNPs in or near genic regions, we utilize the classification scheme that dbSNP employed to define a SNP's “functional state”: SNPs in coding sequences can be either nonsynonymous or synonymous; within an intron, mRNA UTR, or within 2 kb of a gene (locus region), or it could be a change in an intron/exon splice junction. However, because genes can be transcribed from either sequence strand (the plus or minus strand), some SNPs may not belong to a single, unique category. SNPs belonging to more than one category are indicated as being in “multiple classes” and are generally within genes, but fall into more than one functional classification. If no functional information is present in dbSNP (i.e., if the SNP is more than 2 kb from a known dbSNP gene), we defined the SNP to be “nongenic.”

We calculated the frequencies of SNPs in the HapMap (HapMap Public Release #16a) (HapMap, http://www.hapmap.org), the Perlegen database (as of June 15, 2005) (Perlegen, http://genome.perlegen.com), and in the 100K set in the categories described above, defined by the position of the SNPs relative to genic and nongenic regions. To examine the distribution of SNPs relative to the chromosomal density of the arrayed variation, we plotted: 1) the 100K set–SNP density versus the HapMap density of markers for each chromosome; and 2) the density of SNPs on the 100K GeneChip Arrays against the proportion of intergenic and coding SNPs.

The amount of information extracted by the 100K SNP set was quantified using a framework for measuring the amount of missing data described elsewhere [9]. The framework can be used for measuring association/correlation between two data types (e.g., genotype and haplotype data for a set of markers) when the interest is in testing case-control differences for the complete/ideal data type (e.g., haplotype frequencies). The measures of information are defined as a function of asymptotic relative efficiencies which are, for given alternative hypotheses, ratios of sample sizes of the two data types necessary to achieve the same power. For example, given a set of markers for which we would like to test case-control differences in haplotype frequencies, the framework can be used to measure the effect of unknown haplotype phase on power; the corresponding measure shows the ratio of number of haplotype pairs versus unphased genotypes sets that lead to the same power.

In this paper we are interested in the effect of genotyping a subset of the genomic variation on the power of association studies and we can quantify this by measuring the amount of information the set of genotyped variation has on the untyped variation. Because we are interested in local patterns, we constructed information contents for each SNP separately. For each “target” marker in the HapMap SNP set, *T,* we use the framework to define the measure, *M_D_*(*T,*A) that quantifies how much information for estimating its allele frequencies is available in the group, A, of markers in the 100K SNP set that are in the neighborhood of *T*. In the situation of one “target” marker, the measure can be viewed as a multilocus measure of linkage disequilibrium, and is numerically equal to measures based on the coefficient of determination [14,15]. The properties and interpretation of *M_D_* are identical to the ones of *r* ^2^ [16]. In particular, the measure is between 0 and 1, equal to 0 when *T* and A are in linkage equilibrium, and to 1 when the unphased genotypes in A can be used to determine the genotypes at *T*. The measure is a function of haplotype frequencies in the population of interest, frequencies that are estimated from a reference database such as HapMap.

There are only 120 independent haplotypes available in the CEU and YRI datasets of HapMap, and this leads to practical constraints in calculating *M_D_*(*T,*A) (in theory, A contains all the markers in the 100K set). The estimation of haplotype frequencies and functions of them, such as *M_D_,* is not reliable for rarer haplotypes that occur when a large number of SNPs spread over longer distances is used. For this reason, we decided to use at most four 100K Array markers in A, the set that is used to indicate the information on *T*. To avoid inflating the LD values because of overfitting, we only use markers located within 200 kb of *T*. We constructed an algorithm that finds the most informative (highest *M_D_* values) markers (at most four) within the specified region (200 kb) of *T;* the markers are not necessarily consecutive in the physical ordering of the 100K SNP set.

## Abbreviations

100K set: Affymetrix GeneChip Human Mapping 100K SNP array set
CEPH and CEU: Utah residents with ancestry from northern and western Europe
LD: linkage disequilibrium
MAF: minor allele frequency
SNP: single nucleotide polymorphism
YRI: Yoruba people in Ibadan, Nigeria
